# Supplementary material for: Dopamine builds and reveals reward-associated latent behavioral attractors
Source: Nat Commun. 2024 Nov 13;15:9825. doi: 10.1038/s41467-024-53976-x (PMC11561151; doi:10.1038/s41467-024-53976-x)
Supplement: Supplementary file 1 — Supplementary Information [file 41467_2024_53976_MOESM1_ESM.pdf]

Supplementary Materials

Supplementary Methods related to the model

Supplementary Figures and Legends : Figures S1, S2, S3, S4, S5

Supplementary References

Supplementary methods related to the model

e-mouse navigation

In MAGNet, e-mouse navigation was modeled, in a circular arena (radius r_{arena}), as a process where orientation and speed were governed by a convergence toward either a default objective that consisted in approaching and aligning with the arena wall (answering to a need for security), or a goal-directed objective, answering to a need for exploration, the discovery and the retrieval of rewarded locations (i.e. circles with radius r_{reward}). While the default behavior was set according to ballistic laws in the model, goals were driven by population dynamics of the recurrent neural network (see below).

The mouse position was denoted $P = \{X_P, Y_P\}$, with X_P and Y_P its cartesian coordinates. The position vector was

$$\vec{P} = \left(X_P\ Y_P\right) = d_P\left(cos(\theta_P)\ sin(\theta_P)\right) \text{ (1)}$$

with $d_P = ||\vec{P}||$ the distance to the center of the arena O and $\theta_P = (\widehat{\vec{P}, \vec{i}})$ the directional angle of the position vector ($\vec{i}$ =(1, 0)). The mouse moved according to

$$\vec{V} = \frac{d\vec{P}}{dt} = \left(\frac{dX_P}{dt}\ \frac{dY_P}{dt}\right) = V_P\left(cos(\theta_V)\ sin(\theta_V)\right) \text{ (2)}$$

where V_P was the linear speed and $\theta_V = \left(\widehat{\vec{V}, \vec{i}}\right)$ the direction of movement, i.e. the directional angle of the mouse speed vector, termed hereafter the speed angle.

e-mouse linear speed dynamics

The e-mouse linear speed obeyed

$$\tau_V \frac{dV_P}{dt} = F_{V_D} + F_{V_G} \text{ (3)}$$

where the terms $F_{V_D} = V_D - V_P$ and $F_{V_G} = V_G - V_P$ modeled the contribution of default (subscript D) and goal behaviors (subscript G) to linear speed.

On the one hand, F_{V_D} drove linear speed toward the default command speed V_D, which was expressed as

$$V_D = V_{max} L(d_D, \sigma_D) A\left(\Delta\theta_D\right) \quad (4)$$

where V_{max} was the maximal linear speed, $L(d, \sigma) = exp(-\frac{d^2}{2\sigma^2})$ and $A(\theta) = \frac{1+cos\,(\theta)}{2}$ respectively denote exponential colinear (with characteristic distance σ) and cosine angular tuning functions for motor commands [1], d_D the distance separating the e-mouse and the default objective D, and $\Delta\theta_D = \theta_V - \theta_D$ the angular difference between the speed and default objective angles.

At each time, D was defined as the nearest point from e-mouse's position situated on a circle concentric with the circular arena wall with $r_d = r_{arena} - r_{mouse}$, with r_{arena} the arena radius and r_{mouse} the e-mouse body's half width, i.e. at the nearest possible distance from the wall, when considering the physical dimension of the e-mouse body. The default objective angle was computed as $\theta_D = \left(1 - L\left(d_D, \sigma_D\right)\right)\theta_P + L\left(d_D, \sigma_D\right)\theta_T$, where θ_P was the directional angle from the animal position P to its projection onto the wall D, and θ_T was the directional angle tangential to the arena circular wall at point D and in the direction of e-mouse movement.

Overall, F_{V_D} modeled the propensity of e-mouse to be driven by the default command speed V_D, which was important when the e-mouse was 1) approaching the arena wall and heading toward it (typically small d_D (yielding $\theta_D \sim \theta_P$) and $\theta_V \sim \theta_P$, resulting in substantial $L(d_D, \sigma_D)$ and $A\left(\Delta\theta_D\right)$ values) and 2) aligning parallel to the arena wall (typically $d_D \sim 0$

(yielding $\theta_D \sim \theta_T$) and $\theta_V \sim \theta_T$, resulting in large $L(d_D, \sigma_D)$ and $A(\Delta\theta_D)$ values). Conversely, the contribution of the default behavior to the e-mouse overall speed vanished when the e-mouse was far from, or not aligned with, the arena wall.

On the other hand, F_{V_G}, drove the e-mouse linear speed toward the goal command speed V_G, which was expressed as

$$V_G = V_{min} + (V_{max} - V_{min})[(1 - L(d_G, \sigma_G))A(\Delta\theta_G) + L(d_G, \sigma_G^{away})A(\Delta\theta_G + \pi)] \quad (5)$$

where V_{min} was the e-mouse's minimal linear speed, d_G the distance separating the e-mouse and goal objective (hereafter denoted as the internal goal) G, $\Delta\theta_G = \theta_V - \theta_G$ the angular difference between the speed angle and $\theta_G = (\widehat{\vec{PG}, \vec{i}})$ the directional angle from the e-mouse to the internal goal. Altogether, F_{V_G} modeled the propensity of the e-mouse to be driven by the goal command speed, which was important when the e-mouse was 1) far from the internal goal and heading toward it (large d_G so $(1 - L(d_G, \sigma_G)) \sim 1$ and $\theta_V \sim \theta_G$ such that $A(\Delta\theta_G)$ is large), or 2) nearby the internal goal and moving away from it (small d_G so $L(d_G, \sigma_G^{away}) \sim 1$ and $\theta_V \sim \theta_G + \pi$ such that $A(\Delta\theta_G + \pi)$ is large). The scaling of linear tuning functions, when moving toward or away from the internal goal were determined by σ_G and σ_G^{away}, with $\sigma_G < \sigma_G^{away}$ so that navigation was faster when escaping away from a recently visited rewarded point. This hypothesis was necessary to avoid otherwise inevitable (although unrealistic) e-mouse repeated navigational loops at rewarded locations.

The internal goal G was determined according to a probabilistic soft-max process with G drawn, at each time-step, from the normalized exponential probability distribution

$$p\left(G = P_{TF\,(j)}\right) = \frac{exp(\beta_{SM} f_j)}{\sum_k exp(\beta_{SM} f_k)} \quad (6)$$

where $P_{TF\,(j)} = \left(X_{TF\,(j)}\, Y_{TF\,(j)}\right)$ was the preferred position and f_j the estimated firing frequency of neuron j, β_{SM} the inverse temperature of the process and k indexing neurons taking part in the soft-max. The estimated firing frequency was obtained by filtering spiking with an exponential kernel with time constant τ_F. Preferred positions were organized on a square lattice following the X and Y axes that covered the arena, with 70% of neurons within the arena and 30% outside on X and Y axes. Neurons' preferred positions covered a surface area more than twice that of the arena, so that the internal goal could lay outside the arena. The soft-max was thus computed with neurons whose preferred positions were closer than $r^{max}_{soft-max}$, so that G essentially laid within the arena. This ensured that goal-directed influence balanced the centrifugal influence of the default behavior, such that a naïve (i.e. before learning) e-mouse spent ~60% of their time in the default behavior (i.e. running along walls). Convergence to the internal goal could nevertheless tend to drive the e-mouse outside the arena sometimes. To avoid this unrealistic behavior, the distance of the e-mouse to the arena center, d_P, was reset to r_d when this happened.

e-mouse angular dynamics

The e-mouse angular direction θ_V obeyed

$$\tau_{\theta_V} \frac{d\theta_V}{dt} = (1 - \frac{V}{V_{max}})(F_{\theta_D} + F_{\theta_G}) \quad (7)$$

with the first term catching the slower rotation of animals when moving faster, and F_{θ_D} and F_{θ_G} represented contributions of default and goal behaviors to e-mouse orientation changes.

Rotation speed toward the default objective was governed by

$$F_{\theta_D} = L(d_D, \sigma_D)\Delta\theta_D \quad (8)$$

so that it was larger when θ_V was far from θ_D and when the e-mouse approached arena walls ($L\left(d_D, \sigma_D\right)\sim 1$), which insured a progressive rotation toward θ_T (i.e. the e-mouse aligned with the wall when approaching). Rotation was essentially independent of the default behavior far from the wall, being instead mostly goal-directed, with rotation toward the internal goal obeying

$$F_{\theta_G} = A(\Delta\theta_G)\Delta\theta_G \quad (9)$$

where rotational speed scaled with the difference between e-mouse's direction θ_V and θ_G the angle facing the internal goal, but only when the e-mouse was essentially influenced by goals situated in its visual foreground landscape ($A\left(\Delta\theta_G\right)$ vanished at large $\Delta\theta_G$ values). This hypothesis, which expressed a visual gating of internally-guided behaviors, reduced the noise of goal-directed navigation but was not essential to the results.

Pause and redirection behaviors

The e-mouse had behavioral pauses (during which rotational or linear speed was null) that occurred spontaneously with increasing probability when closer to the arena wall, as in real mice. Pause times where thus drawn according to a Poisson process with a rate scaled with the distance to the center of the arena: $\frac{d_P}{r_{arena}}\lambda_{Pause}$, each pause lasting d_{Pause}. Redirections of the e-mouse occurred at the end of pauses, by drawing the new angular direction from a von Mises distribution [2] with mean θ_V and concentration κ_{redir} (i.e. with a circular standard deviation of $\frac{\pi}{4}$). In order to avoid unrealistic redirections toward the exterior of the arena

when at its edges, directions were redrawn when $\vec{PO}.\frac{d\vec{P}}{dt} < 0$ (centrifugal redirection) with probability $p_{redraw} = L(d_D, \sigma_D)$ (nearby 1 in the close vicinity of the arena wall).

Local recurrent neural network biophysical model

We built a biophysical model of a prefrontal local recurrent neural network, endowed with detailed biological properties of its neurons and connections (*3*). The network model contained N neurons that were either excitatory (E) or inhibitory (I) (neurons projecting only glutamate or GABA, respectively), with probabilities p_E and $p_I = 1 - p_E$ respectively and $\frac{p_E}{p_I} = 4$ [3]. Connectivity was sparse (i.e. with probability connection p_C [4]), with no autapse (self-connections). Synaptic weights $w_{(i,j)}$ of existing connections were initiated with a value μ_w, before possible consecutive additional Hebbian assemblies were learnt or written by hand (see below).

To cope with simulation times required for the massive explorations of the model, neurons were modeled as leaky integrate-and-fire (LIF) neurons. The membrane potential of neuron j obeyed

$$\{C\frac{dV_{(j)}}{dt} =- (I_{L(j)} + I_{Syn.Rec(j)} + I_{Syn.FF(j)})\ V_{(j)} > \theta_{(j)} \rightarrow V_{(j)} = V_{repol} \quad (10)$$

where was V_{rest} the repolarization potential. The action potential (AP) threshold $\theta_{(j)}$ was adaptive in excitatory neurons, with spike-induced instantaneous increase and exponential convergence with time constant τ_θ toward its steady-state value θ_0:

$$\frac{d\theta_{(j)}}{dt} = \frac{\theta_0 - \theta_{(j)}}{\tau_\theta} + \Delta\theta\ \delta\left(t - t_{(j)}\right) \quad (11)$$

where δ represents the Dirac function and $t_{(j)}$ AP times in neuron j.

The leak current followed

$$I_{L(j)} = g_L\left(V_{(j)} - V_L\right) \quad (12)$$

with g_L the leak conductance and V_L its equilibrium potential.

The recurrent synaptic current on postsynaptic neuron j, from either excitatory or inhibitory presynaptic neurons (indexed by i), was

$$I_{Syn.Rec(j)} = \sum_i \left(I_{AMPA(i,j)} + I_{NMDA(i,j)} + I_{GABA_A(i,j)} + I_{GABA_B(i,j)}\right) \quad (13)$$

The delay for synaptic conduction and transmission, Δt_{syn}, was considered uniform across the network [5]. Synaptic recurrent currents followed

$$I_{x(i,j)} = \overline{g_X} w_{(i,j)} p_{x(i)}\left(V_{(j)} - V_x\right) \quad (14)$$

Where $\overline{g_X}$ was the maximal conductance, $w_{(i,j)}$ was the synaptic weight, $p_{x(i)}$ the opening probability of channel-receptors and V_x the reversal potential. The NMDA current followed specific dynamics

$$I_{NMDA(i,j)} = \overline{g_{NMDA}} w_{(i,j)} f_{DA}^{NMDA} p_{NMDA(i)} x_{NMDA}\left(V_{(j)}\right)\left(V_{(j)} - V_{NMDA}\right) \quad (15)$$

accounting for the voltage-dependence of the magnesium block [6] which was modeled as

$$x_{NMDA}(V) = \left(1 + \left[Mg^{2+}\right]e^{-0.062\,V}/3.57\right)^{-1} \quad (16)$$

and f_{DA}^{NMDA} represented the dopamine-dependent gating of NMDA conductance [7,8] through D1-receptors, affecting equally all synapses of the network (diffuse VTA dopamine input), according to

$$f_{DA}^{NMDA} = f_{DA\,min}^{NMDA} + (f_{DA\,min}^{NMDA} - f_{DA\,max}^{NMDA}) \frac{1}{1+e^{-(x_{DA}-x_{DA}^{NMDA})/k_{DA}^{NMDA}}} \quad (17)$$

where $f_{DA\,min}^{NMDA}$ and $f_{DA\,max}^{NMDA}$ set minimum and maximal gating and x_{DA}^{NMDA} and k_{DA}^{NMDA} were the half-activation and inverse slope of DA concentration sigmoidal effect.

AMPA and GABAA channel rise times were approximated as instantaneous [5] and bounded, with first-order decay

$$\frac{dp_{x(i)}}{dt} = -\frac{p_{x(i)}}{\tau_x^{decay}} + \Delta p_x \left(1 - p_{x(i)}\right) \delta\left(t - t_{(i)}\right) \quad (18)$$

where $t_{(i)}$ represented the pre-synaptic APs' times. In order to account for the longer NMDA [9] and GABAB [10] channel rise times, opening probabilities followed second-order dynamics

$$\left\{ \frac{dq_{x(i)}}{dt} = -\frac{q_{x(i)}}{\tau_x^{rise}} + \Delta q_x \left(1 - q_{x(i)}\right) \delta\left(t - t_{(i)}^k\right) \; \frac{dp_{x(i)}}{dt} = -\frac{p_{x(i)}}{\tau_x^{decay}} + \alpha_x q_{x(i)} \left(1 - p_{x(i)}\right) \right. \quad (19)$$

Recurrent excitatory and inhibitory currents were balanced on average in post-synaptic neurons [11]according to driving forces and excitation/inhibition weight ratio, through

$$\left\{ \overline{g_{GABA_A}} = g_{GABA_A} \frac{-\left(\langle V\rangle - V_{AMPA}\right) p_{E\to X} p_E}{\left(V_{mean} - V_{GABA_A}\right) p_{I\to X} p_I} \; \overline{g_{GABA_B}} = g_{GABA_B} \frac{-\left(V_{mean} - V_{AMPA}\right) p_{E\to X} p_E}{\left(V_{mean} - V_{GABA_B}\right) p_{I\to X} p_I} \right. \quad (20)$$

with $\langle V\rangle = \frac{\left(\theta_0 + V_{rest}\right)}{2}$ an approximation of the average membrane potential, and X the excitatory or inhibitory identity of the postsynaptic neuron receiving the inhibitory current.

The feed-forward synaptic current $I_{Syn.FF(j)}$ – putatively arising from sub-cortical and/or cortical inputs – consisted of an AMPA current

$$I_{Syn.FF(j)} = \overline{g_{AMPA_{FF}}} \, p_{AMPA.FF} \left(V_{(j)} - V_{AMPA}\right) \quad (21)$$

where $p_{AMPA.FF}$ was the sum of two components,

$$p_{AMPA.FF} = p_{Ext} + p_{FB} \quad (22)$$

The first one, p_{Ext}, corresponded to network-wide AMPA inputs from external sources for every network neuron, and built as the convolution by an exponential kernel k_{Ext} (time constant τ_{AMPA}) of a random stochastic process drawn from the normal distribution, with mean and standard deviation derived from the binomial distribution of the number of input

spikes per time step when considering n_{Ext} external independent inputs projecting onto the network and a spiking probability x_{Ext} for each input per given time step:

$$\{m_{Ext} = \Delta p_{AMPA.FF}\, n_{Ext}\, x_{Ext}\ \sigma_{Ext} = \Delta p_{AMPA.FF} \sqrt{n_{Ext}\, x_{Ext}\left(1 - x_{Ext}\right)} \quad (23)$$

The second component, p_{FB}, corresponded to the putatively hippocampal feedback encoding the e-mouse position (Fig. 1E), with neuron j receiving an input current proportional to the activation $k_{FB}\,(j)$ of their preferred position. Activation function were modeled as bivariate distributions centered on the preferred position $(X_{RF\,(j)}, Y_{RF\,(j)})$.

$$k_{FB\,(j)} = \frac{1}{\sqrt{2\pi}\,\sigma_{TF}} e^{-\frac{1}{4}\left(\frac{\left(X_P - X_{TF\,(j)}\right)^2 + \left(Y_P - Y_{TF\,(j)}\right)^2}{\sigma_{TF}^2}\right)} \quad (24)$$

which displayed similar, but flatter profiles, compared to bivariate distributions, to insure a more homogeneous feed-back activation of neurons encoding the e-mouse position and, as a consequence, smoother and more stable learning (see below). Activation function width was determined by σ_{TF}. Finally, the feed-back opening probability was $p_{FB} = k_{FB}\, x_{FB}$, with x_{FB} a constant.

Synaptic plasticity

We built a constrained biochemical model of the pathways' architecture implicated in the dopaminergic reinforcement of synaptic plasticity. Network excitatory synapses underwent a dopamine (DA)-modulated form of Hebbian Spike Timing-Dependent Plasticity (STDP), with pre- then post-synaptic spike sequences leading to potentiation (and post- then pre-synaptic spike sequences depression). Spiking activity patterns did not translate into immediate effective synaptic changes, but rather resulted in synaptic tags, called eligibility traces [12,13], which were read out at the time of dopamine release [14]. Standard Hebbian synaptic STDP rules devoid of reinforcement gating would strengthen any e-mouse navigation

trajectory associated with a chain of neuronal activation. By contrast, in the presence of DA-reinforced plasticity, network synapses are only modified if they have participated in rewarded trajectories.

At the molecular scale, the spike timing-dependence of synaptic plasticity [12,15] was considered to arise from synaptic calcium dynamics in the postsynaptic button [3,16]. Specifically, calcium was computed as

$$Ca = Ca_0 + Ca_{pre} + Ca_{post} \quad (25)$$

which took into account calcium the sum of calcium contributions arising from pre- and postsynaptic spiking. Presynaptic calcium dynamics followed

$$\frac{dCa_{pre}}{dt} = \Delta Ca_{pre} \sum_i \delta\left(t - t_{(i)} - t_D\right) - \frac{Ca_{pre}}{\tau_{Ca}} \quad (26)$$

which modeled the calcium influx due to pre-synaptic spiking through Voltage-Dependent Calcium Channels (VDCCs), with ΔCa_{pre} the calcium step per action potential (AP), $t_{(i)}$ APs' times, and t_D the delay necessary for AMPA channels' activation and excitatory postsynaptic potential (EPSP) buildup driving VDCCs' opening), in addition to extrusion/buffering of this calcium source, with time constant τ_{Ca}. Postsynaptic calcium dynamics followed

$$\frac{dCa_{post}}{dt} = \Delta Ca_{post} \sum_j \delta\left(t - t_{(j)}\right) + \xi_{PrePost} Ca_{pre} \sum_j \delta\left(t - t_{(j)}\right) - \frac{Ca_{post}}{\tau_{Ca}} \quad (27)$$

which took into account extrusion/buffering (last term) in addition to the calcium influx from post-synaptic back-propagated spiking opening VDCCs (first term) and NMDA channels (second term). The NMDA calcium influx was scaled by an interaction coefficient $\xi_{PrePost}$ and depended on the product of the presynaptic calcium contribution and postsynaptic spiking, to account for the associative opening of NMDA channels due to magnesium blockade.

Intracellular calcium activated calcium-dependent kinases and phosphatases (putatively, CaMKII kinase and calcineurin) that competed to form molecular traces [12,17,18], i.e. eligibility traces [14], and which were distinct for potentiation (eLTP) and depression (eLTD) processes [12]. These traces putatively competed for the phosphorylation of an ERK tag [17], which would decay to a non-phosphorylated state if not consolidated by dopamine into effective – reinforced – changes in synaptic weights. In the model, each eligibility trace followed first-order dynamics, i.e.

$$\frac{de}{dt} = K_e(1 - e) - P_e e \quad (28)$$

where kinase and phosphatase activation followed

$$\{K_e = K_e^{max}\frac{Ca^{nH_e}}{Ca_{h,Ke}^{nH_e}+Ca^{nH_e}}\ P_e = P_e^{max}\frac{Ca^{nH_e}}{Ca_{h,Pe}^{nH_e}+Ca^{nH_e}} \quad (29)$$

with K_e^{max} and P_e^{max} the maximum rates, $Ca_{h,Ke}$ and $Ca_{h,Pe}$ the half-activation calcium values and nH_e the Hill coefficient

Experimental studies indicate that the activation of D1 receptors by dopamine increases cAMP levels and, consequently, protein kinase A (PKA) activity [7,19,20], resulting in the transformation of eligibility traces into effective – reinforced – synaptic changes [14], i.e. modified glutamate receptor densities or phosphorylation levels, e.g. through CREB-induced protein synthesis [17]. In the model, excitatory synaptic weights w evolved according to a dopaminergic gating of a kinase/phosphatase cycle activated by e_{LTP} and e_{LTD} eligibility traces [12], with first-order (soft-bound) kinetics :

$$\frac{dw}{dt} = f_{DA}^{STDP} h\left(K_w\left(w_{max} - w\right) - P_w w\right) \quad (30)$$

with w_{max} corresponded to the maximal synaptic weight, f_{DA}^{STDP} the dopamine-gated functional fraction of the kinase/phosphatase cycle and h a variable accounting for

homeostatic synaptic regulation required only for online learning simulations (see below). Kinase and phosphatase activations followed

$$\begin{cases} K_w = K_w^{max} \frac{e_{LTP}^{nH_w}}{e_{h,K}^{nH_w} + e_{LTP}^{nH_w}} \\ P_w = P_w^{max} \frac{e_{LTD}^{nH_w}}{e_{h,P}^{nH_w} + e_{LTD}^{nH_w}} \end{cases} \quad (31)$$

with K_w^{max} and P_w^{max} the maximum rates, $e_{h,K}$ and $e_{h,P}$ the half-activation eligibility values and nH_w the Hill coefficient.

The dopaminergic gating of synaptic plasticity operated on all synapses (diffuse VTA dopamine input) through D1-receptors [7,14,19], and followed

$$f_{DA}^{STDP} = \frac{1}{1+e^{-(x_{DA}-x_{DA}^{STDP})/k_{DA}^{STDP}}} \quad (32)$$

where x_{DA}^{STDP} and k_{DA}^{STDP} were the half-activation and inverse slope of DA concentration sigmoidal effect on plasticity.

Dopamine dynamics

The dopamine concentration, following spontaneous or reward events (at time t_{DA}), obeyed second-order dynamics

$$\begin{cases} \frac{dr_{DA}}{dt} = -\frac{r_{DA}}{\tau_{DA}^{rise}} + \Delta_{DA}\delta\left(t - t_{DA}\right) \\ \frac{dp_{DA}}{dt} = \frac{-p_{DA} + \alpha_{DA} r_{DA}}{\tau_{DA}^{decay}} \\ x_{DA} = x_{DA}^{min} + p_{DA} \end{cases} \quad (33)$$

where τ_{DA}^{rise} and τ_{DA}^{decay} were rise and decay time constants, x_{DA}^{min} the minimum DA concentration, and α_{DA} a parameter scaling the influence of r_{DA} on p_{DA} dynamics and adjusted to get a maximal value x_{DA}^{max}. Spontaneous events were drawn according to a Poisson process with a rate λ_{rwd}^{sp}, with a refractory period d_{rwd}^{sp}. Reward events occurred when the e-mouse entered a rewarded location. In simulations with three rewarded locations, following consecutive visits of the same location were not rewarded.

Numerical procedures and parameters

MAGNet was simulated and explored using custom developed MATLAB code, whose differential equations were numerically integrated using the forward Euler method ($\Delta t = 1ms$). Most simulations, achieved in offline conditions (Fig. 2), were achieved with the following set of standard parameter values: space and navigation: $\tau_V = 500\,ms$, $\tau_{\theta_V} = 50\,ms$, $V_{max} = 1\frac{m}{s}$, $V_{min} = 0.1\frac{m}{s}$, $\sigma_D = 0.0005\,m$, $\sigma_G = 0.1\,m$, $\sigma_G^{away} = 0.2\,m$, $\lambda_{pause} = 0\,s^{-1}$, $d_{pause} = 0.5\,s$, $\kappa_{redir} = 0.83$, $d_{rwd}^{sp} = 0.5\,s$, $\lambda_{rwd}^{sp} = 0.25$, $r_{arena} = 0.5\,m$, $r_{rwd} = 0.06\,m$, $r_{mouse} = 0.02\,m$, $X_{TF} = Y_{TF} = (-\,0.7308{:}\,0.077{:}\,0.7308)$; neural decoding into the internal goal : $\beta_{SM} = 1.5$, $\tau_F = 100ms$; neural encoding of the e-mouse position: $\sigma_{RF} = 0.075\,m$, $x_{FB} = 0.075$; network architecture: $N = 500$, $p_E = 0.8$, $p_C = 0.75$, $\Delta t_{syn} = 1ms$, $\mu_w = 0.1$, $\sigma_w = 0$, $w_{max} = 5$; intrinsic properties, $C = 1\,\mu F.cm^{-2}$, $\underline{g}_L = 0.05\,mS.cm^{-2}$, $V_L = -70\,mV$, $\theta_0 = -50\,mV$, $\Delta\theta = 50\,mV$, $\tau_\theta = 50\,ms$, $V_{repol} = -60\,mV$; recurrent currents: $\underline{g}_{AMPA} = 0.03\,mS.cm^{-2}$, $\underline{g}_{NMDA} = 0.24\,mS.cm^{-2}$, $g_{GABA_A} = 0.03\,mS.cm^{-2}$, $g_{GABA_B} = 0.0003\,mS.cm^{-2}$, $V_{AMPA} = V_{NMDA} = 0\,mV$, $V_{GABA_A} = -70\,mV$, $V_{GABA_B} = -90\,mV$, $\left[Mg^{2+}\right] = 1.5\,mM$, $\tau_{AMPA}^{decay} = 2.5\,ms$, $\tau_{NMDA}^{rise} = 4.65\,ms$, $\tau_{NMDA}^{decay} = 75\,ms$, $\tau_{GABA_A}^{decay} = 10\,ms$, $\tau_{GABA_B}^{rise} = 90\,ms$, $\tau_{GABA_B}^{decay} = 160\,ms$, $\alpha_{NMDA} = 0.275\,ms^{-1}$, $\alpha_{GABA_B} = 0.015\,ms^{-1}$, $\Delta p_{AMPA} = \Delta q_{NMDA} = \Delta p_{GABA_A} = \Delta q_{GABA_B} = 0.1$; feed-forward currents: $p_{Ext} = 0$,

$\underline{g}_{AMPA.FF} = 0.2\, mS.cm^{-2}$, $f_{Ext} = 25\, Hz$, $n_{Ext} = 30$, $\Delta p_{AMPA.FF} = 0.1$; calcium dynamics: $Ca_0 = 0.1\, \mu M$, $\tau_{Ca} = 50\, ms$, $\Delta Ca_{pre} = \Delta Ca_{post} = 0.5\, \mu M$, $\xi_{PrePost} = 6.5$, $t_D = 20\, ms$; synaptic weight plasticity: $K_w^{max} = 0.15\, ms^{-1}$, $P_w^{max} = 0.03\, ms^{-1}$, $e_{h,K} = 0.25$, $e_{h,P} = 1$, $nH_w = 4$; eligibility traces : $K_{eLTP}^{max} = P_{eLTP}^{max} = K_{eLTD}^{max} = P_{eLTD}^{max} = 0.04\, ms^{-1}$, $Ca_{h,KeLTP} = 1.65\mu M$, $Ca_{h,PeLTP} = 0.495\mu M$, $Ca_{h,KeLTD} = 1.25\, \mu M$, $Ca_{h,PeLTD} = 0.375\, \mu M$, $nH_e = 4$; dopamine properties: $x_{DA}^{min} = 0.1$, $x_{DA}^{max} = 1.1215$, $\tau_{DA}^{rise} = 100\, ms$, $\tau_{DA}^{decay} = 500\, ms$, $\Delta_{DA}^{rwd} = 1$, $\Delta_{DA}^{sp} = 0.25$, $x_{DA}^{STDP} = 0.225$, $k_{DA}^{STDP} = 0.005$, $f_{DA_{min}}^{NMDA} = 0.1$, $f_{DA_{max}}^{NMDA} = 1$, $x_{DA}^{NMDA} = 0.125$, $k_{DA}^{NMDA} = 0.005$.

Initial conditions and simulation setups

The model was initialized with randomized membrane potentials (uniformly distributed in $\left[\theta_0, \theta_0 - 5\right] mV$) and synaptic channel openings mimicking average channel openings ($p_{AMPA} \sim 0.0025$, $p_{NMDA} \sim 0.2$, $p_{GABA_A} \sim 0.0025$, $p_{GABA_B} \sim 0.15$), as well as with e-mouse at initial random positions at distance $d_P = 0.75 r_d$, null linear speed and random initial direction θ_V.

Online learning simulations (Fig. 2d) lasted 300 seconds and consisted of 10 concatenated simulations of 30 seconds termed sessions, with behavioral pauses when rewarded, without redirection. Online learning dynamics easily yielded saturated synaptic weights and neural activity, even with slower learning kinetic parameters ($K_w^{max} = 0.015\, ms^{-1}$, $P_w^{max} = 0.003\, ms^{-1}$). Such plasticity/activity runaway is a classical issue when assessing online learning in random recurrent networks. It arises from the positive

feedback linking excitatory activity and plasticity between excitatory neurons and is likely stabilized by different homeostatic processes providing counteracting negative feedback at the neuronal and network scales. In the present decision architecture, this problem was largely amplified by the additional positive feedback linking connectivity and neural activity, on the one hand, and e-mice behavior, on the other hand. For instance, increased connectivity at rewarded locations (Hebbian assemblies) increased reward rates, which in turn increased DA-reinforcement of STDP at these Hebbian assemblies. In the context of the present study, we found that synaptic homeostasis was essential in the online learning setup (Fig. 2d). Therefore, synaptic homeostasis was constrained by distinct homeostatic processes at excitatory synapses, which were required in online simulations, but not offline simulations (Fig. 2e-i; see below). Hence, in addition to a hard-bound $w_{max} = 5$, excitatory-excitatory synapses underwent synaptic scaling, which normalizes synaptic connections. We considered a form of synaptic scaling that included both presynaptic and postsynaptic normalization, i.e.,

$$w_{(i,j)}(t + \Delta t) = w_{(i,j)}(t)\frac{\sum_{i=1}^{n_E} w_{(i,j)}(t_0)}{\sum_{i=1}^{n_E} w_{(i,j)}(t)}\frac{\sum_{j=1}^{n_E} w_{(i,j)}(t_0)}{\sum_{j=1}^{n_E} w_{(i,j)}(t)} \quad (34)$$

which allowed a limit to catastrophic plasticity/activity runaway eventually occurring at synapses linking neurons of Hebbian assemblies and the rest of the network. In addition, we considered two forms of saturation constraining plasticity runaway. First, by assuming that no plasticity occurred at spiking post-synaptic frequencies superior to a critical frequency set as $f_h = 1/D$, i.e. putatively through presynaptic calcium saturation. This process helped avoiding plasticity/activity runaway within each Hebbian assembly. Second, by assuming that the total amount of post-synaptic potentiation admits a maximum within each neuron, putatively due to upstream resources availability (e.g. pool of precursors limiting the

synthesis of new glutamatergic receptors). This process constrained the spatial extension of Hebbian assemblies. Altogether, these saturations terms wrote

$$h = H\left(f < f_h\right)\left(1 - \frac{\sum_{i=1}^{n_E} \Delta w^{+}}{\Delta w^{+}_{max}}\right) (35),$$

with H the Heaviside function, $\Delta w^{+}_{max} = n_E \mu_w$ the maximal amount of possible potentiation changes and $\sum_{i=1}^{n_E} \Delta w^{+}$ hard-bound limited by Δw^{+}_{max}. Although not crucial for offline learning (Fig. 2e-i, 3), these homeostatic processes were kept in that case, for the sake of simplicity. In a similar vein, because getting strong and compact Hebbian assemblies during online learning proved difficult under certain modeling and parameter choices, we found easier to set constant eligibility time constants (which otherwise non-linearly depended on the calcium activation of their kinase/phosphatase cycles $\tau_e = 1/(K_e + P_e)$), with $\tau_{eLTP} = \tau_{eLTD} = 250ms$. For the sake of simplicity, this option was also kept for offline learning, although not essential in that setup (Fig. 2e-i, 3).

In offline simulations (Fig. 2e-i), average performance rates were computed over 10 simulations in each of 18x18 conditions DA-excitability and DA-plasticity. Simulations lasted 60 seconds, with a pause rate $\lambda_{Pause} = 1/3\ s^{-1}$. Distance, speed and performance were averaged over simulations in each condition. For each simulation, distance between rewards was calculated as the total traveled distance divided by the number of rewards, maximum speed as the average of maximum speeds between consecutive rewards in portions of the travel done within the arena (i.e., d_P<0.45; e-mice had an arbitrarily higher default speed when running along walls) and performance as the number of rewards divided by simulation duration. Fig. 2h and 2i were derived from map offline simulations by computing the average

and standard mean error of maximum speed after a reward and of the duration between two rewards (computed as the inverse of the performance rate). Specifically, curves in Fig. 2h were computed by averaging across vertical slices of data in the map of Fig. 2f and the inverse of data in the map of Fig. 2g for DA plasticity parameter values in the range 3.5-5. Curves in Fig.2i were obtained by averaging these data across horizontal data for DA excitability parameter values in the range 0.7-1. In Suppl Fig. 4a, maximal speed was computed as the mean of its value in the map of Fig. 2f for DA-Plasticity parameter in the range 3.5-5 and DA-excitability in the range 0.7-1 (Reinforcement + Spontaneous DA), DA-Plasticity 3.5-5 and DA-excitability 0-0.3 (Reinforcement only), DA-Plasticity 0-1.5 and DA-excitability 0.7-1 (No reinforcement + Spontaneous DA) and DA-Plasticity 0-1.5 and DA-excitability 0-0.3 (no DA).DA-excitability was parameterized with $f_{DA\ max}^{NMDA}$ in the interval $= [0, 1]$. DA-plasticity was mimicked by initiating the connectivity matrix before model simulation, as if plasticity had previously built three Hebbian assemblies (i.e. there was no plasticity during offline simulations). This initialization consisted in adding three Hebbian assemblies centered at rewarded locations. Each of these bivariate gaussian Hebbian assemblies consisted of the synaptic matrix w_k, built as the auto-association (i.e. external product) of a vertical vector specifying gaussian distance of each neuron preferred position to rewarded location k (with spatial standard deviation $\sigma_{rwd} = 0.125\ m$), i.e.

$$w_k^{(i,j)} = v_k^i v_k^j \quad (36)$$

with

$$v_k^l = \frac{1}{\sqrt{2\pi}\,\sigma_{rwd}} e^{-\frac{1}{2}\left(\frac{\left(X_k - X_{TF(l)}\right)^2 + \left(Y_k - Y_{TF(l)}\right)^2}{\sigma_{rwd}^2}\right)} \quad (37)$$

and $P_k = \left(X_k\, Y_k\right)$ is the position of reward location k. In Fig. 3d, we only kept synapses oriented toward each reward locations within Hebbian assemblies (i.e. $w_{(i,j)} = 0$ when $w_{(j,i)} > w_{(i,j)}$) to assess a scheme where STDP would have yielded purely asymmetric connections. The Hebbian assemblies were then normalized to have a maximal weight w_{max} in the interval $[0, 5]$, and added to a constant mean μ_w

$$w_{(i,j)} = \mu_w + \sum_{k=1}^{3} \frac{w_{max}}{max(w_k)} w_k \quad (38).$$

Offline learning simulations (Fig. 3b) consisted of 100 successive learning trials lasting 1.5 seconds. To speed up simulations, e-mice were initialized with $d_P = 0.3\, m$, heading toward the central rewarded location and with maximum linear speed. In Fig. 3d-f, h, simulations lasted 2.5 seconds, with the e-mouse initialized randomly in the arena, with maximum linear speed and random initial direction θ_V. In Fig. 3f, the angular speed was slower by a factor 10 before phasic DA, so that the e-mouse displayed equivalent positions at that time.

Realizations of von Mises distributions were numerically computed using the code developed by D. Muir [21].

Behavioral Potential Energy (BPE) theory

In order to better understand how e-mouse behavior arises from past dopaminergic reinforcement (DA-plasticity) and online motivational dopaminergic modulation (DA-excitability), we built a simplified theory capturing essential causal and dynamical traits governing the full decision architecture model. To reduce dimensionality, we consider that, thanks to revolution symmetry in the one rewarded location setup, spatial behavior is reducible to one dimension, with rewarded location set at position $p_{AH} = 0$. Also, the theory

is built as a simplified representation of e-mouse navigation that neglects the detailed dynamics of linear and angular speed ballistic commands considered in the model. In particular, the contribution of the default behavior to linear speed, which is negligible at a certain distance from the arena walls in the model, is not taken into account. Moreover, we focus on how e-mouse navigation depends on the essential interactions linking p, the e-mouse position encoded by feed-forward hippocampal inputs to the network resulting in a bump of neural activity (see e.g. Fig. 1e, lower panel, top maps), and g, the internal goal position decoded downstream by basal ganglia through soft-max computation.

This theoretical framework illustrates how goal-directed mouse behavior can be interpreted in the framework of attractorial dynamics within a landscape of behavioral potential energy (BPE), which depends on both DA-plasticity and DA-excitability. Building the theory unraveled two mechanisms driving e-mouse navigation. The first mechanism relates to the local positional stability of the activity bump, in the vicinity of the Hebbian assembly (HA). The second mechanism acts at a global scale of the arena and depends on the neural activity at the HA. Thus, in the theory, we posit that e-mouse p and DA dependent goal-directed navigation obeys a velocity law including the two mechanisms

$$\frac{dp}{dt} = v(p, DA) = v_{local}(p, DA) + v_{global}(p, DA) \quad (39).$$

In the following, we first assess how each term can be described in a reduced and tractable fashion from the model dynamics. We then show how BPE can be derived and interpreted.

The first, local, mechanism relates to the positional stability of the activity bump. In the case when no HA is present (i.e., before reward-place learning; e.g. Fig. 2B, trial #1, left panel), both feed-forward inputs encoding the e-mouse position and recurrent connections are symmetric with regard to position p, such that the activity bump displays a symmetric spatial firing frequency around p. As a result, g, the decoded internal goal position, which

statistically reflects the position of the activity bump maximum, is also situated at p. Hence, the e-mouse is on its goal, convergence is already achieved and there is no movement.

By contrast, let's consider the case where a HA is present (due to previous place-reward association; Fig. 2B, trial #100, left panel), with the e-mouse in the vicinity of the HA. In such a situation, within the activity bump, excitatory synaptic currents generated in excitatory neurons closer to the HA, by excitatory neurons farther from the HA (centripetal currents), are larger than centrifugal currents generated at reciprocal synapses. This is due to the fact that centripetal currents occur at synapses with larger synaptic weights (i.e., higher in the HA weight gradient), compared to centrifugal currents. The resulting firing frequency profile of the activity bump is biased toward the HA, so that, on average, the decoded internal goal position, g, lies closer to the HA, compared to p. As p converges toward g, it continuously moves in the direction of the HA. In turn, as p is moving toward the HA, so too does the activity bump and its soft-max readout, g. Altogether, the weight gradient yields an attractorial convergence of the activity bump, g and p toward the HA. This convergence will obviously be stronger near the HA, where the synaptic gradient is steeper. Moreover, the large increase in NMDA currents mediated by DA (DA-excitability) will strongly amplify the gradient of excitatory currents due to DA-plasticity (i.e., the difference between centripetal and centrifugal currents) and the subsequent attractorial convergence toward the HA, through a *deepening* of the HA attractor, as unraveled by the theory (see below and Fig. 2G-I). Accordingly, the local mechanism to e-mouse velocity scales with the gradient of excitatory currents

$$v_{local}(p, DA) = \alpha_w \frac{dI_{Exc}(p,DA)}{dp} \quad (40)$$

where α_w is a constant and $I_{Exc}(p, DA)$ is the DA-dependent excitatory current received by excitatory neurons at position p, which can be approximated as

$$I_{Exc}(p, DA) = \overline{g_{AMPA}} w(p) p_{AMPA}\left(V_{bump} - V_{AMPA}\right) + \overline{g_{NMDA}}(DA)\, w(p) x_{NMDA}(V_{bump}) p_{NMDA}\left(V_{bump} - V_{NMDA}\right)$$

(41)

where $\overline{g_{AMPA}}$ and $\overline{g_{NMDA}}$ are maximal conductances with $\overline{g_{NMDA}}$ depending on DA-excitability, $w(p)$ the sum of incoming synaptic weights on the neuron with preferred position centered at position p, $x_{NMDA}(V_{bump})$ is the non-linear activation of NMDA channels at the mean bump membrane potential V_{bump} and where p_{AMPA} and p_{NMDA} gating variables at firing frequency f_{bump} can be obtained by steady state approximation from equations 18 and 19 :

$$\{p_{AMPA} \sim \Delta p_{AMPA} \tau^{decay}_{AMPA} f_{bump}\ p_{NMDA} \sim \Delta q_{NMDA} \alpha_{NMDA} \tau^{rise}_{NMDA} \tau^{decay}_{NMDA} f_{bump} \quad (42).$$

Therefore,

$$I_{Exc}(p, DA) = w(p) \hat{I}_{Exc}(DA) \ (43)$$

with

$$\hat{I}_{Exc}(DA) = \left(\overline{g_{AMPA}} \Delta p_{AMPA} \tau^{decay}_{AMPA}\left(V_{bump} - V_{AMPA}\right) + \overline{g_{NMDA}}(DA)\, x_{NMDA}(V_M) \Delta q_{NMDA} \alpha_{NMDA} \tau^{rise}_{NMDA} \tau^{decay}_{NMDA}\left(V_{bump} - V_{NMDA}\right)\right) f_{bump}$$

(44)

is the DA-dependent current per weight unit at firing frequency f_{bump}. Note that $\hat{I}_{Exc}(DA)$ is an inward current, i.e., algebraically negative, which yields the sign of the local contribution to velocity and Behavioral Potential Energy (equations 40 and 56, see below). Note also that inhibitory currents can be neglected, as they display no spatial weight gradient in the model. The local mechanism contribution thus writes

$$v_{local}(p, DA) = \alpha_w \frac{dw}{dp}(p)\, \hat{I}_{Exc}(DA) \ (45)$$

and depends on both DA-plasticity (i.e., on the weight gradient) and DA-excitability (i.e., DA-modulated NMDA current in the activity bump).

The second mechanism acts at the global spatial scale and also emerges from the interaction of DA-plasticity and DA-excitability: it arises from the DA-dependent increase of NMDA currents within the HA itself. Generally, the internal goal g is detected at p because the activity bump is the strongest spot of activity in the network. However, in the presence of DA, the increase of NMDA currents is boosted by large HA weights, which triggers massive associative co-activation of neuronal activity in the HA. Therefore, g almost instantaneously switches to 0, the AH position (see Fig. 2F center panel and Fig. 2H center column). Note that, due to noise in network dynamics within model simulations, activity can still be higher at p than at g in a number cases, accounting for why g does not always converge to the HA (Fig. 2H center column). When acting, this mechanism operates at the global scale of the whole arena, independent of the position of the e-mouse (by contrast to the first mechanism, which acts locally in the vicinity of the HA). Thus, it yields attractorial convergence of the activity bump toward the HA through a widening of the HA attractor (as opposed to attractor deepening in the local mechanism), as shown below (see Fig. 2G-I).

So, the global velocity contribution writes

$$v_{global}(p, DA) = \alpha_g(g(DA) - p) \quad (46)$$

Here, for the sake of simplicity, we use a crude linear dependence of the distance of the e-mouse to the internal goal. However, using more complex dependance reminding model ballistics – or even zero order dependance – would yield qualitatively similar results. The essential point here is that, as shall be seen below, BPE increases with distance in all these cases. Moreover, based on simulations, we set $g(p, DA = 0) \sim p$ when DA is absent. By contrast, when DA is present, decoded internal goal position g, is statistically essentially detected at either one of the two higher spots of activity in the network, i.e., the activity bump (p) and the HA (p_{AH}), with probabilities related to their respective spiking frequency.

Approximately, the internal goal position g can thus be estimated to lie, on average, at the barycenter of both spots weighted by their spiking activity

$$g(DA = 1) \sim \frac{f_{bump} p + f_{AH} p_{AH}}{f_{bump} + f_{AH}} \quad (47),$$

when DA is present. In that case, the global velocity therefore writes

$$v_{global}(p, DA = 1) = \alpha_g \frac{f_{AH}}{f_{bump} + f_{AH}} \left(p_{AH} - p\right) \quad (48)$$

Setting

$$\rho \equiv \frac{f_{AH}}{f_{bump} + f_{AH}} \quad (49)$$

and leveraging on the fact that $p_{AH} = 0$ leads to

$$v_{global}(p, DA = 1) = - \alpha_g \rho p \quad (50)$$

when $DA = 1$. Lumping both cases ($DA = 0$ and $DA = 1$) is possible by writing:

$$v_{global}(p, DA) = - \alpha_g \rho DA p \quad (51)$$

Overall, the velocity law governing e-mouse p and DA dependent goal-directed navigation is thus

$$v(p, DA) = v_{local}(p, DA) + v_{global}(p, DA) = - \alpha_w \frac{d}{dp} w(p) \hat{I}_{Exc}(DA) - \alpha_g \rho DA p \quad (52)$$

In the case where both DA plasticity and DA-excitability are present and the e-mouse is in the vicinity of the HA, the local and global terms hypothesize distinct positions of g, i.e., at p or p_{AH}, respectively. However, in that case, p_{AH} and p are practically almost confounded in the context of the noisy chaotic activity of the network. Moreover, and as a consequence, the global effect is minute, compared to the local effect, which is overwhelming. We therefore kept this crude formulation for the sake of simplicity, without developing a more complex description taking into account which term has to be considered in which case (presence or not of DA-plasticity and DA-excitability, distance to the HA).

The potential of any one dimensional dynamical system

$$\frac{dx}{dt} = f(x) \quad (53)$$

can be computed as

$$E_P = - \int f(x)dx \quad (54),$$

based on the physical idea of the potential energy [22]. We therefore define the Behavioral Potential Energy (BPE) of the e-mouse at each point as

$$E_P^{behavior}(p, DA) = - \int v(p, DA)dp \quad (55),$$

which yields

$$E_P^{behavior}(p, DA) = \alpha_w w(p)\hat{I}_{Exc}(DA) + \frac{1}{2}\alpha_g \rho DAp^2 + Cst \quad (56),$$

where Cst is an integration constant.

This expression captures how, at a previously rewarded location, reinforced HA weights induce attractorial dynamics though both their profile, whose gradient locally destabilizes the activity bump, and their strength, which shifts the internal goal at the global scale. Moreover, this expression accurately accounts for how shape, width and depth of the Hebbian-based attractor depend on previous DA reinforcement (DA-plasticity), current DA motivation (DA-excitability) and their interaction, and how it acts depending on e-mouse position. In doing so, it offers a framework for interpreting coupled dynamics between collective network activity at the activity bump and the HA, the e-mouse position, and the internal goal. Specifically, it mechanistically accounts for weak convergence to the – latent – attractor at the previously rewarded location under DA-plasticity alone (Fig. 2F-I, left), deepening and widening of attractorial convergence under DA-plasticity + DA-excitability (Fig. 2F-I, center and Fig. 2H) and the absence of attractorial convergence under DA-excitability (Fig. 2F-I, right).

BPE was computed using a gaussian-shaped distribution of weights

$$w(p) = w_{min} + (w_{max} - w_{min})e^{-\frac{1}{2}\left(\frac{p}{\sigma_w}\right)^2} \quad (57)$$

centered at position 0 and with spatial standard deviation σ_w. For purely illustrative purpose in Fig. 2, a phenomenological term was added to BPE to account for short-distance attraction to arena walls due to the default behavior:

$$E_P^{default}(p) = -\alpha_d\left(e^{-\frac{1}{2}\left(\frac{(p+r_d}{\sigma_d}\right)^2} + e^{-\frac{1}{2}\left(\frac{(p-r_d}{\sigma_d}\right)^2}\right) (58),$$

but this term is not part of the theory by itself. Regarding display specificities, in Fig. 2I, one-dimensional BPE was integrated in the range $\left[r_{-arena}, r_{arena}\right]$, using integration constants chosen so that $E_P^{behavior}\left(-r_{arena}, DA\right) = E_P^{behavior}\left(r_{arena}, DA\right) = 0$ in each condition (DA-plasticity, DA-excitability, DA-plasticity + DA-excitability). In Fig. 2H, BPE (left column) was generated in two dimensions from one-dimensional BPE (Fig. 2I) by revolution symmetry, for the sake of illustration, i.e., visual correspondence with model simulations (center and right columns). BPE contour levels correspond to $BPE = -[0\ 0.05\ 0.1\ 0.2\ 0.4\ 0.6\ 0.8\ 1]$. Theory parameters were as following: $w_{min} = 0.1$, $w_{max} = w_{min}$ in the DA-excitability conditions and $w_{max} = 3$ in DA-plasticity and DA-plasticity + DA-excitability conditions. The firing frequency $f_{bump} = 16\,Hz$ was derived from simulations. The mean voltage at the peak of the activity bump was also taken from simulations: $V_{bump} = [-32.5, -32.5, -25]mV$ in all conditions (such depolarized values in the bump arise from spiking-induced depolarization of the adaptive AP threshold). The Gaussian widths were $\sigma_w = 0.075m$ and $\sigma_d = 0.01m$. In Fig. 2, we used $\alpha_w = 20C^{-1}m^2$, $\alpha_g = 1.25s^{-1}$, $\alpha_d = 0.025m^2s^{-1}$, but these parameters can be arbitrarily

scaled without any qualitative change in the BPE landscape. We made no specific hypothesis concerning the relative values of firing frequency and considered the parsimonious case where $f_{bump} = f_{AH}$, i.e., $\rho = 1/2$ in Fig. 2. Again, this specific choice had no consequence on the BPE landscape. Other theory parameters were as in model simulations.

Reduced model account of the literature on DA effects on ongoing goal-directed behaviors

We assessed how the MAGNet reduced model could allow interpreting current discrepancies regarding the effect of DA manipulation on action statistics, in terms of its predictions on DA-motivated goal-directed behavior (Fig. 5 and Suppl. Fig. 3). In this framework, navigation to the goal was considered as an action, and obeyed

$$\frac{dx}{dt} = -\frac{dE_P}{dx} \quad (59),$$

as can be inferred from equations (53) and (54). We characterized such actions (i.e., navigating from an initial position to the goal) by computing observables referred as time-to-goal, action rate and action probability. The time-to-goal was the elapsed duration from the initial e-mouse position up to the goal, the action rate was computed as the inverse of time-to-goal, and the action probability, the probability of reaching the reward within a given time limit, here 3 seconds. These observables were systematically computed as a function of the initial position of the animal (expressed as the distance to the goal) and with BPE scaled by phasic DA ranging from 0 ("low DA") to 2 ("high DA") times that of the standard phasic DA value in the MAGNet model.

Supplementary figures and legends

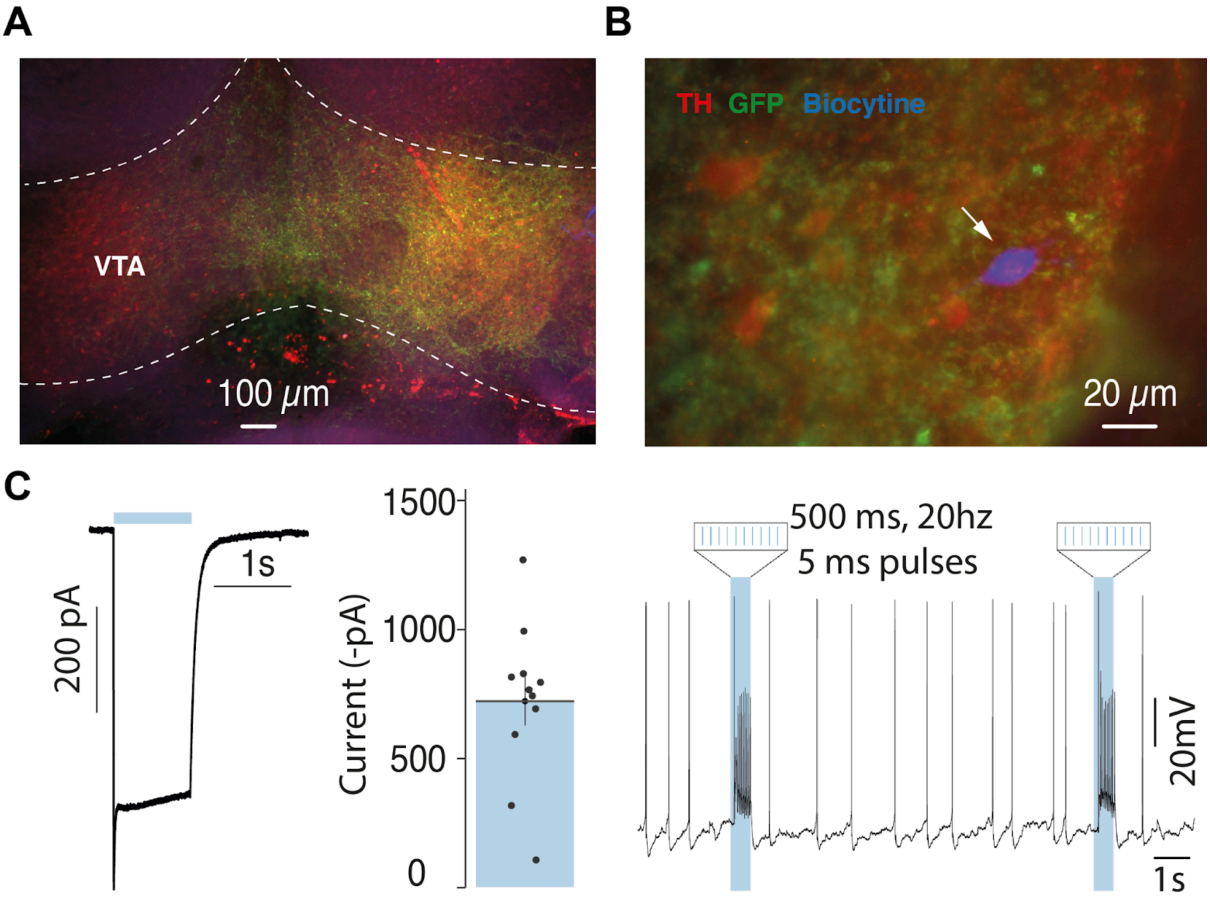

Supplementary Figure 1: Specificity of dopamine control by optogenetics

(**a**) ChR2 was expressed in VTA DAT+ (dopamine) neurons in slices from DAT-Cre mice used for ex-vivo recording. (**b**) Zoom in the example neuron recorded, expressing TH, YFP and filled with biocytin (blue). (**c**) Left, example of current induced by a one second-pulse and average currents from 12 cells, induced by the 10 5ms-pulses at 20Hz. Right, example of bursting driven by 10 5ms-pulses at 20Hz.

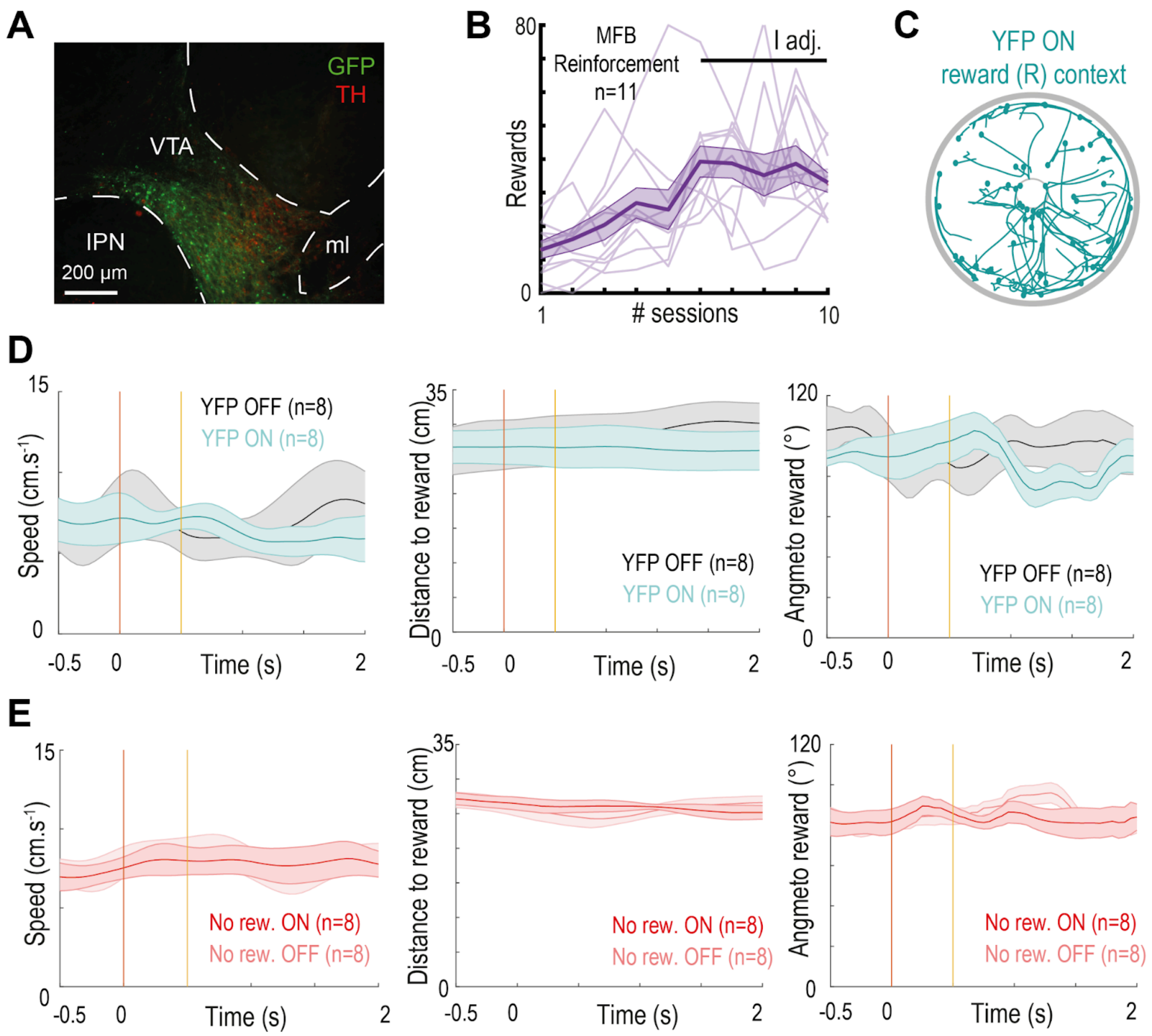

Supplementary Figure 2: Control experiments related to Figure 5.

(**a**) ChR2 was expressed in VTA DAT+ (dopamine) neurons in animals used in Fig. 3 experiments. (**b**) Number of location visits across sessions of MFB reward learning. (**c**) Post-photostimulation bouts of trajectories in the YFP, ON light, R context. (**d**) From left to right: speed (ON versus OFF : $T_{(7)}$=-0.44, p=0.67), distance ($T_{(7)}$=-0.92, p=0.39) and angle ($T_{(7)}$=-0.47, p=0.66) to rewarded location around the time of random VTA photostimulation in the periphery for YFP animals. (**e**) Same as d for Chr2 animals in the "no reward" condition.

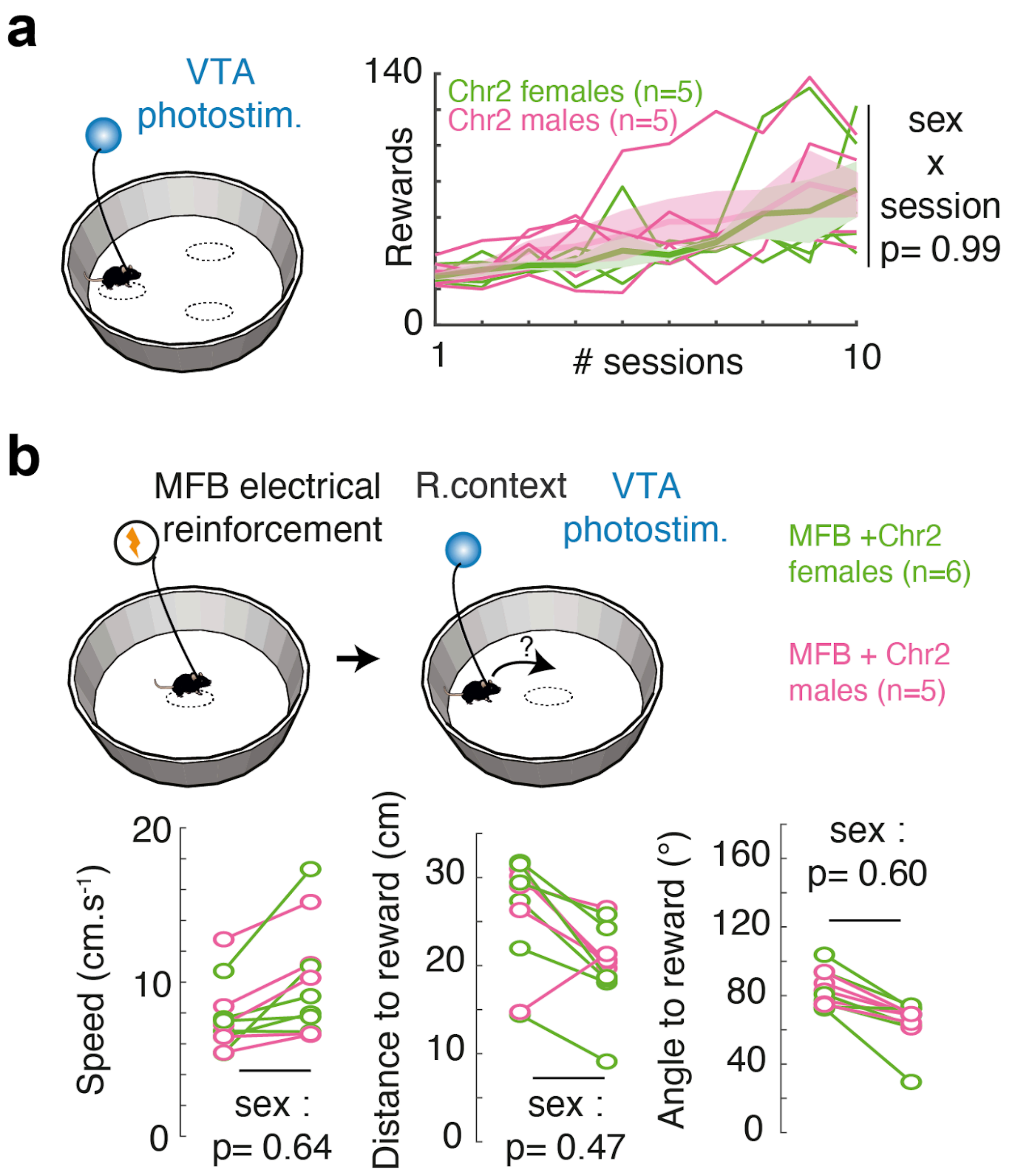

Supplementary Figure 3: Effects of sex on reinforcement and motivation.

(**a**) In the optogenetics reinforcement task (Figure 1), the number of photostimulations against session number for Chr2-expressing (purple) and YFP-expressing (black) animals (same as Fig. 1b) did not differ significantly between female and male mice. (**b**) In the MFB reinforcement-then-VTA photostimulation task (Figure 5), average difference in speed (f), distance to the rewarded location (h), and angle between the animal and the rewarded location (k) between ON and OFF light conditions, in reward ("Chr2") and no reward ("Chr2 No R") contexts, did not differ significantly between female and male mice.

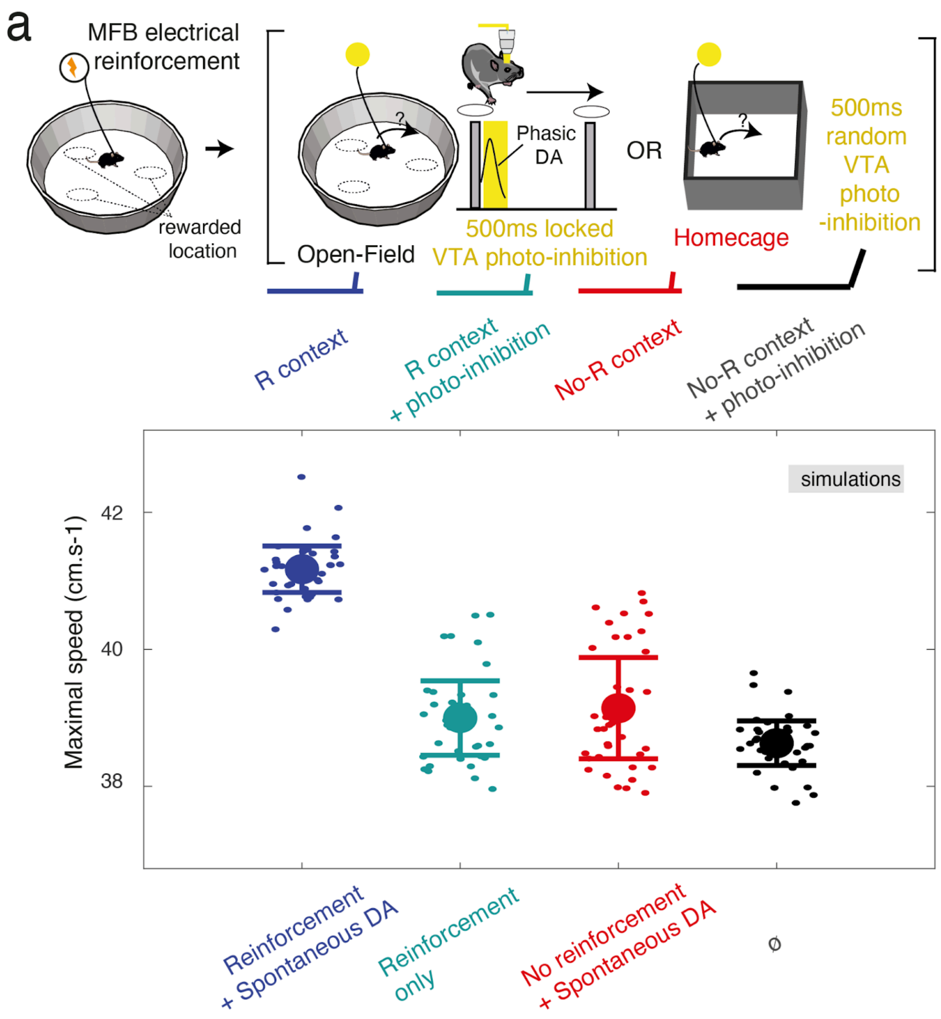

Supplementary Figure 4: MAGNEt simulation of Bousseyrol et al data (VTA photo-inhibition during reward-directed locomotion) .

(**a**) Simulation of Bousseyrol, Didienne et al. reward-directed locomotion task : (top) following MFB electrical reinforcement of three locations in an open-field (electrical equivalent to Figure 1 optogenetics experiment), VTA photo-inhibition at the start of a new trial in the same environment reduces the speed towards the next rewarded location, while random VTA photo-inhibition in the homecage does not affect speed. In the MAGNet framework, these experiments respectively correspond to manipulating DA in the R. context and in the no R. context. (bottom) Simulations of the MAGNet model in these conditions show that inhibiting DA in the R context affects animal speed (blue versus green), but inhibiting DA in the R context does not (red versus black), similar what was found in the experimental data from Bousseyrol, Didienne et al.

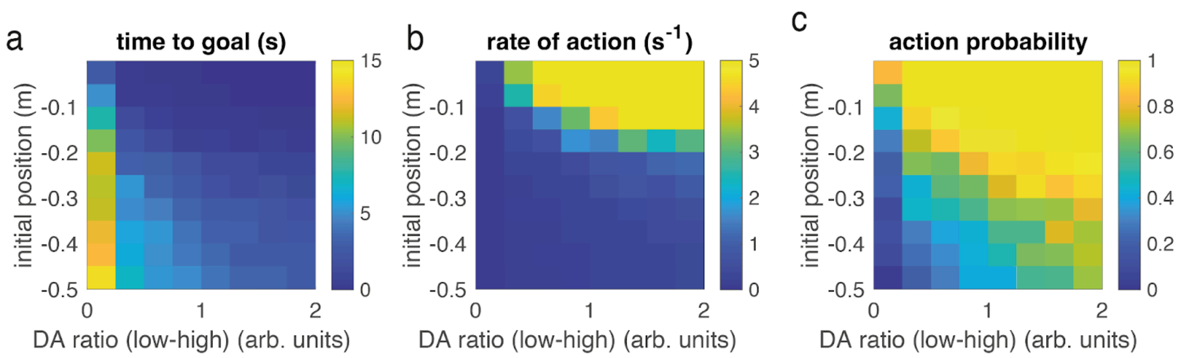

Supplementary Figure 5: Parametric exploration of the reduced model

Time-to-goal, i.e. the elapsed duration between the initial e-mouse position and the reward location (**a**), action rate (inverse of time-to-goal) (**b**), action probability, i.e. the probability that the reward location is attained within trial duration (c) with e-mouse moving according to the reduced model (see Methods), as a function of the initial distance to the reward location and with BPE scaled with DA ranging from 0 ("low DA") to 2 ("high DA") times that of the standard phasic DA value.

Supplementary References

1. Todorov, E. & Jordan, M. I. Optimal feedback control as a theory of motor coordination. *Nat. Neurosci.* **5**, 1226–1235 (2002).
2. Best, D. J. & Fisher, N. I. Efficient simulation of the von Mises distribution. *J. R. Stat. Soc. Ser. C Appl. Stat.* **28**, 152–157 (1979).
3. Sarazin, M. X., Victor, J., Medernach, D., Naudé, J. & Delord, B. Online Learning and Memory of Neural Trajectory Replays for Prefrontal Persistent and Dynamic Representations in the Irregular Asynchronous State. *Front. Neural Circuits* 57 (2021).
4. Thomson, A. M., West, D. C., Wang, Y. & Bannister, A. P. Synaptic connections and small circuits involving excitatory and inhibitory neurons in layers 2–5 of adult rat and cat neocortex: triple intracellular recordings and biocytin labelling in vitro. *Cereb. Cortex* **12**, 936–953 (2002).
5. Brunel, N. & Wang, X.-J. Effects of neuromodulation in a cortical network model of object working memory dominated by recurrent inhibition. *J. Comput. Neurosci.* **11**, 63–85 (2001).
6. Jahr, C. E. & Stevens, C. F. Voltage dependence of NMDA-activated macroscopic conductances predicted by single-channel kinetics. *J. Neurosci.* **10**, 3178–3182 (1990).
7. Tritsch, N. X. & Sabatini, B. L. Dopaminergic modulation of synaptic transmission in cortex and striatum. *Neuron* **76**, 33–50 (2012).
8. Durstewitz, D. & Seamans, J. K. The Dual-State Theory of Prefrontal Cortex Dopamine Function with Relevance to Catechol-O-Methyltransferase Genotypes and Schizophrenia. *Neurodev. Transit. Schizophr. Prodrome Schizophr.* **64**, 739–749 (2008).
9. Wang, H., Stradtman III, G. G., Wang, X.-J. & Gao, W.-J. A specialized NMDA receptor function in layer 5 recurrent microcircuitry of the adult rat prefrontal cortex. *Proc. Natl. Acad. Sci.* **105**, 16791–16796 (2008).
10. Destexhe, A. *et al.* Kinetic Models of Synaptic Transmission. *Methods in neuronal modeling 2 (1998): 1-25.*

11. Xue, M., Atallah, B. V. & Scanziani, M. Equalizing excitation–inhibition ratios across visual cortical neurons. *Nature* **511**, 596–600 (2014).

12. He, K. *et al.* Distinct eligibility traces for LTP and LTD in cortical synapses. *Neuron* **88**, 528–538 (2015).

13. Shindou, T., Shindou, M., Watanabe, S. & Wickens, J. A silent eligibility trace enables dopamine-dependent synaptic plasticity for reinforcement learning in the mouse striatum. *Eur. J. Neurosci.* **49**, 726–736 (2019).

14. Izhikevich, E. M. Solving the distal reward problem through linkage of STDP and dopamine signaling. *Cereb. Cortex N. Y. NY 1991* **17**, 2443–2452 (2007).

15. Bi, G. & Poo, M. Synaptic modifications in cultured hippocampal neurons: dependence on spike timing, synaptic strength, and postsynaptic cell type. *J. Neurosci.* **18**, 10464–10472 (1998).

16. Graupner, M. & Brunel, N. Calcium-based plasticity model explains sensitivity of synaptic changes to spike pattern, rate, and dendritic location. *Proc. Natl. Acad. Sci.* **109**, 3991–3996 (2012).

17. Okuda, K., Højgaard, K., Privitera, L., Bayraktar, G. & Takeuchi, T. Initial memory consolidation and the synaptic tagging and capture hypothesis. *Eur. J. Neurosci.* **54**, 6826–6849 (2021).

18. Magee, J. C. & Grienberger, C. Synaptic plasticity forms and functions. *Annu. Rev. Neurosci.* **43**, 95–117 (2020).

19. Seamans, J. K. & Yang, C. R. The principal features and mechanisms of dopamine modulation in the prefrontal cortex. *Prog. Neurobiol.* **74**, 1–58 (2004).

20. Zhang, J. *et al.* Activation of the dopamine D1 receptor can extend long-term spatial memory persistence via PKA signaling in mice. *Neurobiol. Learn. Mem.* **155**, 568–577 (2018).

21. Dylan Muir (2022). vmrand(fMu, fKappa, varargin) (https://www.mathworks.com/matlabcentral/fileexchange/37241-vmrand-fmu-fkappa-varargin), MATLAB Central File Exchange. Retrieved July 21, 2022.

22. Strogatz, S. H. *Nonlinear Dynamics and Chaos: With Applications to Physics, Biology, Chemistry, and Engineering*. (CRC press, 2018).
